# Sustainable taste: a conceptual integration of sensory, sociocultural, and structural dimensions for interdisciplinary research and practice

**DOI:** 10.3389/fnut.2026.1811381

**Published:** 2026-04-29

**Authors:** Claudia Squarzon, Wesley R. Dean, Barbara Vad Andersen

**Affiliations:** 1 Department of Food and Resource Economics, University of Copenhagen, Copenhagen, Denmark; 2 Sino-Danish College (SDC), University of Chinese Academy of Sciences, Beijing, China; 3 Food Quality Perception and Society Team, iSense Lab, Department of Food Science, Faculty of Technical Sciences, Aarhus University, Aarhus, Denmark

**Keywords:** behavior change, COM-B model, sensory–emotional–relational (SER) framework, food practices, sustainable diets, sustainable food consumption, taste, sustainable taste

## Abstract

The purpose of this paper is to analyze the concept of taste in relation to sustainable food consumption, highlight disciplinary ambiguities in its meaning, and propose a refined definition that supports interdisciplinary dialogue and practical application. We ask: what do we mean by “taste,” and how can it be conceptualized across disciplines to support a shared language for the sustainable food transition? To address this question, we conduct a conceptual analysis spanning sociology, anthropology, nutrition, consumer research, and sensory science. Based on this synthesis, we propose a definition of sustainable taste as a multidimensional construct encompassing sensory, emotional, and relational (SER) dimensions. The Sensory–Emotional–Relational (SER) framework conceptualizes taste not only as a physiological and hedonic experience, but also as a socially and contextually embedded phenomenon shaped through everyday food practices and sociocultural environments. To demonstrate its analytical utility, we integrate the SER framework with the COM-B model of behavior change (Michie et al. 2011). By embedding taste within Capability, Opportunity, and Motivation, we show how taste operates simultaneously as an individual-level sensory experience and as a product of social, material, and economic contexts that structure food choice. Reframing taste as a nexus of sensation, emotion, relation, and context provides a conceptual bridge between behavioral science and practice-oriented approaches to food consumption. This integration offers a foundation for designing interventions and policies that make sustainable diets both appealing and accessible across diverse sociocultural groups.

## 1 Introduction

The urgency of shifting toward sustainable diets has gained increasing recognition over recent decades. The 1994 Oslo Symposium defined sustainable consumption as meeting basic needs and improving quality of life while minimizing resource use, toxic materials, and pollution (1). Since then, global momentum has grown: the United Nations highlights sustainable consumption and production as key to the 2030 Agenda for Sustainable Development (2), and the EAT–Lancet Commission calls for radical dietary shifts to protect human and planetary health (3, 4). Together, these frameworks emphasize that dietary change is central to sustainability.

Within this context, taste occupies a central, yet paradoxical position. It is consistently identified as one of the strongest determinants of food choice (5–14). Yet, its conceptualization and application in empirical studies differs widely across disciplines. The divergent usages can generate conceptual ambiguities and hinder interdisciplinary dialog.

Similar challenges have been observed in other interdisciplinary fields, where conceptual ambiguity initially hindered progress. For instance, early debates on sustainability and resilience were marked by fragmented definitions across disciplines until shared frameworks enabled more coherent research and policy development (15–17). In a comparable way, advancing sustainable dietary transitions requires a clearer and more integrated conceptualization of taste that can bridge disciplinary perspectives and support both theoretical and practical applications.

Against this backdrop, this paper asks the following questions: (1) How is “taste” conceptualized across disciplines in relation to food consumption? (2) What conceptual ambiguities arise from these differing perspectives? (3) How can taste be reconceptualized to better support sustainable dietary transitions? More specifically, the paper explores how an interdisciplinary framework can integrate the sensory, emotional, and relational dimensions of taste to better explain and influence everyday food practices.

Addressing these questions is not only a theoretical exercise but also a practical necessity. Without a coherent understanding of taste, it risks being marginalized in policy and intervention design. By contrast, a multidimensional conceptualization of taste can inform strategies that align sensory pleasure with sustainability goals, thereby increasing the likelihood of behavioral change.

To address these questions, the paper employs a conceptual analysis approach (18–20), drawing on interdisciplinary literature to examine how taste is defined, measured, and applied across fields. This approach enables the identification of key conceptual distinctions, overlaps, and gaps that currently limit integration. The analysis is structured around three dimensions. First, we examine disciplinary contributions to understanding taste in the context of sustainable food consumption, with particular attention to how taste is operationalized in empirical research (e.g., sensory testing, consumer experiments, and sociocultural analyses). Second, we analyze conceptual ambiguities between related constructs such as taste, preference, liking, and flavor, which often complicate interpretation and comparison across studies. Third, we assess how taste is incorporated into behavior-change frameworks and identify limitations in current models.

Building on this analysis, the paper proposes the concept of *sustainable taste*, defined as a multidimensional construct that integrates sensory experience, emotional meaning, and relational context. To operationalize this concept within behavior-change research and practice, we extend the COM-B model (21) by introducing the Sensation–Emotion–Relation (SER) framework. This extension positions taste as a central driver of behavior, rather than treating it as a secondary attitudinal factor.

By developing an integrative conceptualization of taste, this paper aims to contribute to both theory and practice. It advances interdisciplinary dialog by clarifying key concepts and their interrelationships while also providing a foundation for designing interventions that leverage taste as a resource for promoting sustainable diets. In doing so, it responds to growing calls for approaches that connect sensory experience, social practice, and structural conditions in efforts to transform food systems (22–25).

## 2 Conceptual background

The transition to more sustainable diets is recognized as a crucial challenge of our time. Food choice is situated at the heart of these transitions. While production-side changes, such as agricultural innovation and food system restructuring, are essential, they are insufficient without corresponding shifts in consumption patterns. Everyday food practices, what people buy, cook, eat, and dispose directly influence the demand for resource-intensive foods, the adoption of plant-forward diets, and the cultural acceptability of sustainability interventions (26). Thus, understanding the determinants of food choice has become a key area of inquiry for researchers, practitioners, and policymakers seeking to promote sustainable diets.

Among the many factors shaping food choice, including price, availability, convenience, and social norms, taste consistently emerges as one of the strongest predictors (8, 27, 28). Survey-based studies using the Food Choice Questionnaire show that taste is typically ranked as the most important factor guiding food selection, often above health and environmental concerns (29–31). Experimental studies further demonstrate that sensory appeal strongly predicts actual consumption behavior, even when individuals express intentions to eat more sustainably (32–34).

Importantly, taste preferences are not fixed. Evidence from sensory and nutrition research shows that repeated exposure to specific foods can significantly increase liking and acceptance. For example, repeated tasting interventions have been shown to increase preference for vegetables and other initially less-preferred foods over time (35). Similarly, product reformulation studies indicate that improving the sensory qualities of plant-based alternatives enhances their acceptance among consumers (36–38). These findings suggest that taste can be shaped through experience and learning, rather than acting solely as a barrier to dietary change.

Despite its central role, however, taste has been comparatively under-theorized in sustainability research. It is often treated as a background variable or a constraint on behavior, rather than as a complex, multifaceted concept worthy of theoretical elaboration (39). Some studies implicitly assume that sustainable or healthy foods are less palatable, reinforcing the perception that taste and sustainability are in conflict (40). Others reduce taste to subjective liking, overlooking its physiological, emotional, and sociocultural dimensions (41–43).

Such simplifications have important implications. When taste is narrowly or inconsistently defined, interventions tend to focus primarily on information provision or rational decision-making, while neglecting the sensory and experiential aspects that drive everyday food choices. This helps explain why information-based campaigns often have limited impact on behavior, as they fail to engage with the primary motivations underlying food consumption (44–47).

A more comprehensive understanding of taste is therefore necessary. Rather than viewing taste as a fixed individual preference, it should be conceptualized as a multidimensional phenomenon shaped by interactions between sensory perception, individual experience, and sociocultural context. This perspective aligns with interdisciplinary approaches that emphasize the integration of physiological, psychological, and sociocultural dimensions in the study of food behavior (48–50).

## 3 Methods

To unpack the concept of taste in the context of sustainable food consumption, this study adopts a conceptual analysis approach informed by a narrative review. We examine how “taste” is defined, interpreted, and operationalized across disciplines and within the context of sustainable food consumption. The literature was identified through targeted searches in Google Scholar and JSTOR, selected for their broad coverage of interdisciplinary scholarship. Searches were conducted using combinations of keywords related to taste, food behavior, and sustainability. Search strings included, among others: “taste AND sustainability,” “food preference AND sustainable diet,” “dietary choice AND sustainability,” “food practices AND sustainable food consumption, “taste AND sustainable eating,” “food behavior AND sustainability,” “social construction of taste AND sustainable food,” “food preferences AND cultural identity,” and “consumer behavior AND taste.”

The search focused primarily on literature published from 1990 onwards, reflecting the emergence and consolidation of research on sustainability and food systems. However, foundational theoretical contributions that have significantly shaped conceptualizations of taste were also included (51–61).

The search included peer-reviewed journal articles, review papers, and book chapters, as well as selected policy documents and institutional reports (e.g., from the Food and Agriculture Organization and the World Health Organization) that have contributed to shaping contemporary understanding of sustainable diets. The search was limited to publications in English.

Studies were included if they met the following criteria: (i) explicitly addressed taste, food preference, or sensory experience as a determinant of food choice or dietary practices; (ii) examined food consumption in relation to sustainability, culture, or behavioral change; and (iii) represented relevant disciplinary perspectives, including nutrition science, sensory science, consumer research, behavioral science, sociology, anthropology, philosophy, sustainability studies, and behavioral economics. Studies were excluded if: (i) taste was not substantively analyzed (e.g., only briefly mentioned in introductory sections without analytical relevance); (ii) the term “taste” was used metaphorically or figuratively rather than referring to sensory perception, food preferences, or food-related behavior; or (iii) the source was non-scholarly or lacked sufficient methodological transparency. During the screening process, articles were further excluded if taste did not feature in the research questions, analytical framework, or study findings, or if it was employed solely as a cultural or rhetorical metaphor unrelated to food perception or consumption.

The review process followed a structured narrative review approach rather than a formal systematic review protocol, as the goal was conceptual synthesis rather than exhaustive evidence aggregation. The search yielded an ideal pool of approximately 948 records. After removing duplicates and applying initial inclusion criteria (e.g., English language, publication date from 1990 onward except for foundational works), 633 papers were screened by title and abstract. This phase involved three reviewers to ensure consistency and reduce bias. Of those screened, 518 papers met the eligibility criteria based on full-text review. Reasons for exclusion at this stage included lack of empirical data on taste or sustainability, focus outside human dietary behavior, or insufficient methodological rigor. The final set comprised the 77 most relevant papers, including peer-reviewed journal articles, systematic reviews, book chapters with foundational theory, and selected policy/institutional reports. The selection prioritized conceptual relevance, theoretical contribution, and empirical robustness, consistent with the aim of developing an integrative conceptual synthesis rather than exhaustive coverage.

Screening was conducted by the first author, with uncertain cases discussed with the co-authors to ensure consistent application of the inclusion criteria. An overview of the key publications included in the conceptual analysis is provided in Table 1, which summarizes disciplinary perspectives, conceptualizations of taste, and their relevance to sustainable food consumption. The literature was reviewed iteratively to identify recurring conceptual patterns, disciplinary differences, and opportunities for theoretical integration. Data extraction focused on how taste is defined, conceptualized, and operationalized within different disciplinary contexts. For each study, we recorded the disciplinary field, conceptual framing of taste, related constructs (such as preference, liking, flavor, and palatability), and any links made to sustainable food consumption or behavioral change.

**Table 1:** Overview of key literature included in the conceptual analysis.

#	Author(s)	Year	Discipline	Study type	Conceptualization of taste	Link to sustainability	Key contribution
1	Bourdieu	1984	Sociology	Theory	Taste as cultural capital	Indirect	Taste reflects class inequality
2	Bourdieu	1986	Sociology	Theory	Habitus and distinction	Indirect	Structural shaping of preferences
3	Ochs et al.	1996	Anthropology	Ethnography	Socialized taste	Indirect	Taste learned in families
4	Jackson	2005	Geography/Sociology	Empirical	Contextual consumption	Yes	Food environments shape preferences
5	Jackson	2020	Geography/Sociology	Empirical	Contextual consumption	Yes	Environment influences diet
6	Johnston & Szabo	2011	Sociology	Empirical	Food and identity	Yes	Ethical eating and class
7	Goodman et al.	2012	Human Geography	Empirical	Ethical food practices	Yes	Moral food consumption
8	Chen & Nelson	2020	Economics	Empirical	Socioeconomic taste	Yes	Income shapes food choice
9	Monterrosa et al.	2020	Nutrition	Review	Sociocultural taste	Yes	Culture and sustainability
10	Ramos	2023	Social Science	Empirical	Structural taste	Yes	Inequality in diets
11	Kristóf & Megyesi	2024	Sociology	Empirical	Food practices	Yes	Stratified consumption
12	Dean et al.	2012	Consumer Research	Empirical	Preference under constraint	Yes	Cost limits food choice
13	Dean	2023	Consumer Research	Empirical	Economic constraint	Yes	Affordability vs. sustainability
14	Neuman	2019	Sociology	Theory	Embodied taste	Yes	Critique of determinism
15	Becuț & Puerto	2017	Sociology	Empirical	Food practices	Yes	Shifting consumption
16	Fernqvist et al.	2024	Consumer Research	Empirical	Contextual taste	Yes	Real-life context matters
17	Warde	2016	Sociology	Theory	Taste as practice	Yes	Eating as routine practice
18	Symmank	2019	Consumer Research	Empirical	Sensory drivers	Yes	Taste drives choices
19	Fiorentini et al.	2020	Sensory Science	Experimental	Sensory attributes	Yes	Texture/flavor matter
20	Appiani et al.	2023	Food Science	Experimental	Hedonic evaluation	Yes	Acceptance of alternatives
21	Yang & Lee	2019	Consumer Research	Empirical	Perceived taste	Yes	Taste perception affects adoption
22	Hume	1757	Philosophy	Theory	Subjective taste	Indirect	Taste and shared standards
23	Kant	1798	Philosophy	Theory	Esthetic judgment	Indirect	Subjective universality
24	Korsmeyer	1997	Philosophy	Theory	Esthetic taste	Indirect	Taste as evaluation
25	Korsmeyer	2005	Philosophy	Theory	Esthetic taste	Indirect	Food and philosophy
26	Classen et al.	1994	Anthropology	Theory	Sensory culture	Indirect	Cultural senses
27	Howes	2003	Anthropology	Theory	Multisensory	Indirect	Sensory culture
28	Howes	2004	Anthropology	Theory	Multisensory	Indirect	Sensory experience
29	Howes	2009	Anthropology	Theory	Multisensory	Indirect	Cultural perception
30	Howes	2015	Anthropology	Theory	Multisensory	Indirect	Sensory studies
31	Howes	2022	Anthropology	Theory	Multisensory	Indirect	Sensory theory
32	Pink & Howes	2010	Anthropology	Theory	Sensory ethnography	Indirect	Embodied experience
33	Ingold	2011	Anthropology	Theory	Relational perception	Indirect	Situated perception
34	Chau	2008	Anthropology	Empirical	Cultural taste	Indirect	Ritual and taste
35	Hsu	2008	Anthropology	Empirical	Sensory knowledge	Indirect	Cultural shaping
36	Allen-Collinson et al.	2021	Sociology	Empirical	Embodied practice	Indirect	Sensory experience
37	Martikainen & Sakki	2023	Sociology	Empirical	Food discourse	Yes	Meaning-making
38	De Certeau	2011	Sociology	Theory	Everyday practice	Indirect	Consumption practices
39	Shove et al.	2009	Sociology	Theory	Practice theory	Yes	Practices shape behavior
40	Gherardi	2009	Organization Studies	Theory	Practice-based learning	Indirect	Learning through doing
41	Trubek	2008	Anthropology	Empirical	Taste of place	Yes	Geography of taste
42	Sutton	2001	Anthropology	Ethnography	Memory and taste	Indirect	Nostalgia
43	Sutton	2010	Anthropology	Ethnography	Memory and taste	Indirect	Food and memory
44	Højlund & Squarzon	2023	Anthropology	Empirical	Craft and taste	Yes	Skills shape taste
45	Teil & Hennion	2004	Sociology	Theory	Reflexive taste	Indirect	Taste as activity
46	Hennion	2007	Sociology	Theory	Taste as practice	Yes	Relational taste
47	Counihan & Højlund	2018	Anthropology	Edited volume	Food culture	Yes	Social meaning
48	Mondada	2018	Sociology	Empirical	Interactional taste	Indirect	Social coordination
49	De Toffoli et al.	2019	Nutrition	Empirical	Taste perception	Yes	Taste as barrier
50	Liem	2019	Sensory Science	Review	Taste development	Yes	Exposure shapes liking
51	Lourenço et al.	2022	Consumer Research	Empirical	Preference	Yes	Taste vs. sustainability
52	Kershaw et al.	2023	Public Health	Empirical	Food choice	Yes	Taste as determinant
53	Sorlí et al.	2025	Nutrition	Empirical	Taste perception	Yes	Diet adherence
54	Maehle et al.	2015	Marketing	Empirical	Taste expectations	Yes	Expectation effects
55	Andersen et al.	2021	Consumer Research	Empirical	Taste vs. sustainability	Yes	Trade-offs
56	Ballco & Gracia	2022	Economics	Empirical	Consumer preference	Yes	Purchasing behavior
57	Beltrán-De-Miguel et al.	2025	Nutrition	Empirical	Dietary choice	Yes	Taste predicts intake
58	Ajzen	1991	Psychology	Theory	Attitudes	Yes	Planned behavior
59	Kahneman & Tversky	1979	Behavioral Economics	Theory	Decision-making	Indirect	Heuristics
60	Spence	2015	Sensory Science	Review	Multisensory flavor	Yes	Flavor complexity
61	Spence	2022	Sensory Science	Review	Multisensory flavor	Yes	Crossmodal perception
62	Melis & Barbarossa	2017	Consumer Research	Empirical	Liking vs. taste	Yes	Construct differentiation
63	Diepeveen et al.	2022	Sensory Science	Review	Taste vs. flavor	Yes	Measurement clarity
64	Meiselman	1992	Sensory Science	Theory	Contextual eating	Yes	Real-life context
65	Meiselman	2006	Sensory Science	Theory	Contextual eating	Yes	Eating environment
66	Meiselman	2015	Sensory Science	Review	Contextual eating	Yes	Ecological validity
67	Brunsø et al.	2002	Consumer Research	Empirical	Lifestyle	Yes	Context influences
68	Jaeger et al.	2025	Sensory Science	Empirical	Consumer testing	Yes	Limits of lab
69	Cardello & Meiselman	2018	Sensory Science	Empirical	Context effects	Yes	Environment matters
70	Seo	2020	Sensory Science	Review	Multisensory perception	Yes	Contextual effects
71	Rozin & Fallon	1987	Psychology	Theory	Learned taste	Yes	Acquisition
72	Rozin et al.	2008	Psychology	Theory	Moralization	Yes	Disgust/culture
73	Rozin	2015	Psychology	Review	Meaning of food	Yes	Cultural shaping
74	Liberman et al.	2016	Psychology	Empirical	Disgust	Yes	Emotional mechanisms
75	Coulthard et al.	2021	Nutrition	Empirical	Food learning	Yes	Exposure effects
76	Jensen & Lieberoth	2019	Behavioral Science	Empirical	Novel foods	Yes	Cultural acceptance
77	Florença et al.	2022	Food Studies	Empirical	Food acceptance	Yes	Social meaning

The extracted information was then synthesized and organized into analytical matrices corresponding to three selected analytical dimensions, which were subsequently condensed into three tables to provide a structured overview:

1. Disciplinary contributions to understanding taste and sustainability, mapping how different academic fields conceptualize taste in relation to food practices (Table 2).
2. Conceptual ambiguities and overlapping terminologies in taste research, examining how constructs such as preference, liking, flavor, and palatability are used and sometimes conflated across disciplines (Table 3).
3. The role of taste within behavior-change frameworks, assessing how taste is addressed in models of dietary behavior and sustainable food transitions (Table 4).

**Table 2:** Disciplinary contributions to the study of taste in sustainable food consumption.

Discipline/perspective	Key authors/references	Core concept of taste	Methodological approaches	Implications for sustainability
Sociology/social theory	Pierre Bourdieu (53, 54)	Taste as social distinction, cultural capital, and embodied disposition	Surveys, analysis of social practices	Highlights structural constraints on food choice; informs equitable interventions for sustainable consumption
Anthropology/sociology of the senses	Classen et al. (83); Howes (86); Ingold Tim (93); Højlund (106, 153); Teil & Hennion (109), Warde (160)	Taste as relational, performative, and contextually situated; social production of senses	Ethnography, participant observation, *in situ* sensory studies	Supports interventions that leverage social norms and everyday practices to promote sustainable eating
Consumer & sensory science	Meiselman (128, 129); Meiselman, (225); Spence (122, 123); Melis & Barbarossa (121); Jaeger et al. (127)	Taste includes gustatory perception, hedonic evaluation (liking, preference, acceptance)	Laboratory and real-world tasting experiments, choice experiments, self-report measures	Provides evidence for designing foods that are both appealing and sustainable; emphasizes hedonic drivers in behavior change
Psychology/behavioral science	Rozin and Todd (13); Rozin and Fallon (133); Ajzen (117); Kahneman & Tversky (119)	Taste shaped by physiology, learned preferences, socialization, moral and emotional responses (e.g., disgust)	Behavioral experiments, self-report, observational studies	Informs strategies to align sensory pleasure and habit formation with sustainable food behaviors
Nutrition & public health	De Toffoli et al. (110); Liem and Russell (9); Vermeir & Verbeke (226); Sorlí et al. (112)	Taste as individual preference influencing food selection; barrier or facilitator to healthy/sustainable diets	Surveys, controlled interventions, longitudinal studies	Focuses on changing attitudes and preferences to promote sustainable diets through education and policy
Philosophy/esthetics	Hume (57); Kant (58); Korsmeyer (80, 81); Howes (224)	Taste as evaluative judgment bridging subjectivity and normative claims	Conceptual analysis, critical theory	Provides framework for considering social negotiation of taste and shared norms in sustainable food culture
Structural-environmental/economics	(71); Dean, (70); Jackson (64); Jackson et al., (107); Johnston & Szabo (65); Goodman et al. (63)	Taste as shaped by economic and material constraints (“tastes of necessity”)	Quantitative surveys, socio-economic data analysis	Highlights socio-economic barriers to diverse, sustainable diets; informs policy and food access interventions

**Table 3:** Conceptual ambiguities in the use of “taste” and related terms.

Term / concept	Definition/core meaning	Disciplinary emphasis	Measurement/methods	Relevance to sustainable food consumption
Taste	Basic gustatory perception (sweet, sour, salty, bitter, umami)	Sensory science, psychology	Physiological testing, gustatory assays	Central to food acceptance; can guide formulation of sustainable foods that are palatable
Flavor	Multisensory experience integrating taste, smell, texture, auditory cues	Sensory science, consumer research	In situ tasting, sensory panels	Influences overall hedonic appeal; critical for designing sustainable food alternatives
Preference	Relative ranking or choice among food options	Psychology, consumer research	Choice experiments, ranking tasks	Shapes food selection and adoption of sustainable alternatives
Liking	Hedonic or affective evaluation of specific foods	Psychology, sensory science	Self-report scales, hedonic rating	Predicts acceptance of novel or sustainable foods; guides product development
Palatability	Overall degree of pleasure in consumption, context-dependent	Nutrition, sensory science	Observational studies, controlled feeding experiments	Enhancing palatability can support sustainable diet adherence
Embodied taste/habitus	Taste as socially and culturally shaped disposition, reflecting repeated experiences and social context	Sociology, anthropology	Ethnography, qualitative interviews, practice-based observation	Highlights relational and structural influences; informs interventions beyond individual choice
Practice-based taste	Taste as part of routinized food practices involving materials, skills, and meanings	Sociology, anthropology, environmental studies	Observation of everyday practices, ethnography, participatory methods	Supports sustainable food transitions by targeting social norms, infrastructure, and skills
Structural-economic influences on taste	Taste shaped by availability, affordability, and socio-economic constraints (“tastes of necessity”)	Sociology, economics, public health	Socioeconomic surveys, policy analysis, population studies	Emphasizes need for policy, pricing, and infrastructure interventions to enable sustainable choices
Conceptual ambiguities/overlaps	Terms like taste, flavor, preference, liking, and palatability are sometimes used interchangeably	Across disciplines	Varies; often inconsistent	Clarifying terminology is essential for interdisciplinary research and designing effective sustainable interventions

**Table 4:** Presence and treatment of taste in behavior-change models.

Model/framework	Key focus	Treatment of taste	Limitations for dietary change/sustainability	Representative references
Transtheoretical model/stages of change	Individual behavior change through stages (precontemplation → maintenance)	Taste often included as part of motivation or attitude	Neglects structural, social, and environmental factors; taste treated simplistically	(61, 190)
Theory of planned behavior (TPB)	Attitudes, subjective norms, perceived behavioral control	Taste represented as an attitudinal belief	Reduces taste to a cognitive factor; overlooks sensory, cultural, and social dimensions	(117, 118)
Social cognitive theory (SCT)	Reciprocal interactions among behavior, environment, and personal factors	Taste considered a reinforcement or preference	Underplays structural, routine, and cultural influences on behavior	(51, 187)
Health belief model (HBM)	Perceived risks and benefits influence health behaviors	Taste sometimes considered a barrier	Limited in addressing everyday food practices and hedonic motivations	(189, 228)
Practice theory/nudge/choice architecture	Social routines, default options, behavioral nudges	Taste treated mainly as a lever or incentive	Externalizes taste; does not theorize multisensory, sociocultural dimensions	(192, 227)
Social ecological model (SEM)	Multi-level influences: individual, interpersonal, community, policy	Taste often implicit in motivation or social norms	Integrates environment but rarely theorizes taste multidimensionally	(56, 200, 205)
COM-B model	Behavior emerges from Capability, Opportunity, Motivation	Taste typically part of “motivation”	Captures individual-context interaction, but taste rarely separated into hedonic, physiological, relational dimensions	(21, 203)
SER framework (Sensation–Emotion–Relation)	Integrates sensory, emotional, and relational dimensions of food choice	Taste explicitly theorized as multidimensional: sensory, emotional, social	Framework aims to overcome simplifications in existing models; still emerging	Current study proposal

The analytical process followed an iterative conceptual synthesis. First, selected publications were reviewed to identify how taste and related constructs were defined and operationalized across disciplines. Second, key concepts and analytical categories were coded and compared to identify recurring patterns, overlaps, and disciplinary differences. Third, the extracted insights were synthesized into the three analytical dimensions, which were organized into Tables 2–4 to provide a structured overview of disciplinary perspectives, conceptual terminology, and the treatment of taste within behavior-change frameworks.

These dimensions functioned as a structured analytical lens for synthesizing insights across disciplines. They also helped identify conceptual gaps that currently limit the integration of taste into sustainability frameworks. Building on this synthesis, Section 7.3 proposes an extension of the COM-B model by introducing the SER framework (Sensation-Emotion-Relation), which integrates sensory experience, emotional meaning, and social relations to better account for how taste influences sustainable food practices.

## 4 Disciplinary contributions for understanding taste and sustainability

### 4.1 Sociological and anthropological perspectives: taste as social practice

Pierre Bourdieu’s foundational work positioned taste at the center of social stratification, arguing that cultural preferences are not merely individual choices but socially conditioned dispositions that both reflect and reproduce class structures (53). In this view, taste operates as a form of embodied cultural capital: what and how one eats signals social location, education, and distinction. Empirical studies building on this framework show, for example, that higher socioeconomic groups are more likely to consume organic, plant-based, or ethically labeled foods, while lower-income groups tend to prioritize affordability and satiety, reflecting what Bourdieu describes as a “taste of necessity.”

Structural-environmental perspectives further support this view by demonstrating how taste is shaped by material and socioeconomic conditions. Building on Bourdieu’s concept of a “taste of necessity” (53, 54), research shows that food preferences are strongly influenced by income, access, education, and cultural resources (62–69). Empirical studies further demonstrate that economic constraints limit the diversity of food choices, often leading lower-income groups to prioritize affordability and satiety over variety or sustainability considerations (70, 71). Taken together, these findings underscore that taste is not only a sensory or psychological phenomenon but also structurally mediated, linking individual experience to broader systems of inequality.

Food preferences thus function as markers of identity and vehicles of symbolic boundary-making, linking everyday practices of consumption to broader systems of power and inequality.

However, this perspective has been critiqued for its relative determinism and limited capacity to explain shifts in taste practices, particularly in the context of transitions toward sustainable diets (72–74).

Subsequent scholarship has both built upon and critiqued this framework. Alan Warde argues that sociological analyses of eating have tended to privilege the symbolic and esthetic dimensions of taste, often overlooking the physiological and sensory processes through which taste is experienced (75). This critique is supported by empirical findings showing that sensory attributes such as texture, flavor intensity, and familiarity strongly influence food acceptance, even when foods align with ethical or sustainability values (73, 76–79). Warde therefore calls for a more integrated approach that accounts for both social meaning and embodied experience. This highlights a key tension between sociological explanations centered on social distinction and evidence from sensory and consumer research emphasizing hedonic and perceptual factors. This tension is not new. In philosophy and esthetics, taste has long been treated as a key problem precisely because it sits at the intersection of subjective experience and claims to shared validity (57, 58, 80, 81). While not always directly concerned with food, this tradition foregrounds the evaluative nature of taste and its negotiation between individual perception and collective norms. This perspective highlights a foundational challenge that persists across contemporary research: how to account for taste as both personally embodied and socially structured.

Parallel developments in food anthropology and sensory studies further conceptualize taste as situated, relational, and context-dependent (82–94). Research on everyday practices (95–98) demonstrates how taste emerges through routines, material arrangements, and social interactions. Empirical studies in this tradition show, for instance, how taste preferences are shaped by place (99), memory and nostalgia (100, 101), and practices of production and craftsmanship (102), illustrating how sensory experiences are embedded in cultural and temporal contexts. Taste is therefore not a fixed attribute but a dynamic relation between individuals, materials, and social environments (103–109). This perspective is particularly relevant for sustainability, as it suggests that taste can be reshaped through changing practices, contexts, and social meanings rather than treated as a static barrier.

### 4.2 Sensory, nutrition, psychological, and consumer research perspectives

In contrast, nutrition science, sensory science, and consumer research tend to conceptualize taste at the level of individual perception and evaluation. Within these fields, taste is often operationalized through self-report measures (e.g., Likert-scale liking ratings), sensory tests, and discrete choice experiments, and is frequently treated as a key determinant, or barrier, to adopting healthier or more sustainable diets (7, 9, 110–112). Empirical studies consistently show that taste is one of the strongest predictors of food choice, often outweighing sustainability considerations when the two are perceived to be in conflict (113–116).

This perspective is grounded in rational choice and behavioral models, including the theory of planned behavior (117, 118) and behavioral economics (119), which conceptualize food choice as the outcome of individual preferences, attitudes, and intentions. Sensory science further differentiates between constructs often grouped under “taste,” distinguishing gustatory perception from broader multisensory flavor experiences, and separating these from hedonic evaluations such as liking, preference, and acceptance (120–123). These distinctions are operationalized through experimental designs that isolate specific sensory attributes (e.g., sweetness, bitterness, texture) and measure corresponding hedonic responses (124, 125).

However, this individually oriented framework has been critiqued for its limited ecological validity, as laboratory-based measurements do not always translate into real-world behavior (126–128). Meiselman (128, 129) argues that such approaches fail to capture the contextual complexity of real-life eating, where social interaction, atmosphere, prior expectations, and situational constraints shape sensory experience. Echoing this concern, Fernqvist et al. (73) emphasize the importance of interdisciplinary collaboration and *in situ* research designs to better capture food choice as it unfolds in everyday life. Together, these perspectives point toward a more holistic understanding of taste as situated, relational, and context-dependent.

Field studies and in situ experiments demonstrate that contextual factors, such as social setting, food environment, and prior experience, significantly shape taste perception and food choice, often moderating or overriding stated preferences (130–132). This points to a critical gap between controlled measurements of taste and its lived experience in everyday consumption contexts.

Efforts to bridge these perspectives are reflected in the work of Rozin and others, who conceptualize taste as a multisensory, learned, and meaning-laden experience shaped by both biological predispositions and cultural processes (13, 133, 134). Empirical research demonstrates how food preferences are acquired through repeated exposure, socialization, and emotional associations, including mechanisms such as disgust and moralization (135, 136). For example, studies show how culturally learned aversions or preferences (e.g., toward fermented foods or insects) are not reducible to innate sensory properties but are shaped by social and symbolic meanings (137, 138). This integrative approach aligns with socio-anthropological perspectives while retaining attention to sensory and psychological processes.

Taken together, these perspectives highlight that taste cannot be reduced to either a purely sensory response or a purely social construct. Instead, it emerges from the interaction between sensory perception, individual evaluation, social practice, and structural conditions. However, the literature remains fragmented along disciplinary lines: sociological approaches emphasize structure and meaning but often underplay sensory experience; sensory and consumer research prioritize measurement and prediction but tend to neglect social context; and integrative approaches remain relatively underdeveloped.

This fragmentation limits the ability to effectively incorporate taste into sustainability frameworks, where both sensory acceptability and social meaning are critical for dietary change. Addressing this gap requires conceptual models that integrate these dimensions, moving beyond the dichotomy between individual preference and social structure toward a more relational understanding of taste as simultaneously embodied, contextual, and socially mediated.

## 5 Conceptual ambiguities and overlapping terminologies in taste research

### 5.1 Ambiguities in the use of related terms across disciplines

A central challenge in taste research lies in the inconsistent use of related terms such as *taste*, *flavor*, *liking*, *preference*, and *palatability*. While these concepts refer to distinct phenomena, they are often used interchangeably across disciplines, leading to ambiguity in interpretation and measurement.

In sensory and consumer sciences, these distinctions are clearly defined and operationalized. *Taste* refers to basic gustatory perceptions (sweet, sour, salty, bitter, and umami), while *flavor* denotes a broader multisensory experience integrating taste, smell, texture, and even auditory cues (122, 139). *Liking* captures the hedonic evaluation of food, typically measured using rating scales and captured by terms like acceptance, preference, liking, and satisfaction (120–122, 140, 141), whereas *preference* reflects a comparative choice between alternatives (42, 49). *Palatability* describes the overall pleasure derived from food consumption, integrating sensory and contextual factors (142, 143).

Empirical research demonstrates why these distinctions matter. Studies show that liking does not always predict actual food choice, particularly when contextual constraints such as price, availability, or social norms intervene (144, 145). Similarly, preference measured in controlled experimental settings may differ from behavior in real-world environments (146). For example, consumers may report liking plant-based foods in surveys but choose less sustainable options in practice if those are perceived as more convenient or socially normative. These reflects a well-documented attitude-behavior gap: many consumers express positive views about plant-based or sustainable foods, yet buy or eat less sustainable options when these are easier, cheaper, or more socially typical (14, 147, 148).

Despite these distinctions, consumer research often collapses these terms, treating taste, flavor, preference, and liking as interchangeable constructs (125, 149, 150). This simplification can obscure the mechanisms through which food choices are shaped and limit the comparability of findings across studies.

Sociological and anthropological approaches use the concept of taste in a broader sense, encompassing systems of meaning, identity, and social distinction (151, 152). In this context, taste refers not only to sensory evaluation but also to culturally learned dispositions and social practices. While this broader usage captures important dimensions of food behavior, it can create further ambiguity when compared with the more narrowly defined constructs used in experimental research.

Overall, these differences highlight that terminological ambiguity reflects deeper disciplinary assumptions about what taste represents, whether it is a physiological response, an individual evaluation, or a social practice. Clarifying these distinctions is therefore essential for both theoretical integration and empirical research.

### 5.2 Embodiment, practice, and structural contexts of taste

Beyond terminological differences, contemporary interdisciplinary research converges on the view that taste is best understood as an embodied, situated, and relational phenomenon. Drawing on phenomenology and sociology, the concept of embodiment challenges reductionist accounts by emphasizing that sensory experience is simultaneously biological and socially conditioned (52, 53, 60, 74). From this perspective, the body is not merely a passive receptor of stimuli but a site where sensory pleasure, social history, and cultural meaning intersect.

Building on Bourdieu’s notion of habitus, taste can be understood as a set of embodied dispositions formed through repeated exposure and socialization. These dispositions shape not only what individuals like but also how they perceive, evaluate, and engage with food. Recent sociological and anthropological work extends this view by showing how embodied dispositions mediate between the sensory and the symbolic, linking subjective experience to broader structures of inequality, normativity, and cultural meaning (107, 153–160).

This perspective highlights a key analytical shift: taste is neither located solely “in the body” nor externally imposed, but emerges through a recursive relationship between embodied experience and structured environments. However, this contrasts with dominant approaches in consumer research and sensory science, where taste is more often conceptualized as an individual sensory response rooted in physiological perception, neural processing, and hedonic evaluation (161–163). These fields have developed a fine-grained terminology, distinguishing taste perception, flavor attributes, and hedonic constructs such as liking, preference, and acceptance, enabling precise measurement and experimental control (50, 77, 149, 164, 165).

While this analytical precision is a clear strength, it also reveals a limitation: social context is often treated as an external variable rather than a constitutive element of taste. As a result, interventions grounded in these frameworks frequently target individual-level change, through product reformulation, labeling, or nudging, assuming relatively stable and measurable preferences (166). Yet evidence suggests that increasing liking alone does not necessarily translate into sustained behavioral change when structural constraints and social norms remain unaddressed (145, 167).

At the same time, consumer and sensory science is not monolithic. More recent developments within these fields adopt increasingly holistic and interdisciplinary models that integrate physiological, psychological, and sociocultural dimensions. Researchers such as Köster and Mojet emphasize that food choice is shaped by complex interactions between sensory properties, memory, context, and learned behavior (49, 168, 169, 225). These approaches begin to converge with socio-anthropological perspectives by acknowledging that taste and food behavior cannot be fully understood in isolation from their contexts.

Practice-based approaches further deepen this integrative potential by conceptualizing food consumption not as discrete choices but as routinized activities involving materials, meanings, and skills (170). From this perspective, taste is enacted through practices such as shopping, cooking, and eating, rather than existing as a purely internal state.

Accordingly, taste itself can be understood as a practice, better captured as “tasting,” that unfolds through situated, embodied activities. Empirical studies illustrate this dynamic: repeated exposure through cooking and shared meals can reshape preferences over time; participation in collective eating settings (e.g., family meals or workplace canteens) influences what is perceived as acceptable or desirable; and hands-on engagement in food preparation or production (e.g., gardening or artisanal practices) alters sensory appreciation and evaluation. Participation in shared food environments, such as family meals, workplace canteens, or community settings, thus shapes both exposure and preference formation, reinforcing the idea that taste evolves through doing rather than merely sensing (107, 153).

Crucially, these practices are embedded within broader structural and economic conditions. Access to resources, food environments, and pricing structures significantly influence what foods are available, normalized, and desirable. Limited access to fresh or diverse foods can constrain the development of preferences, reinforcing patterns shaped by affordability and availability (171, 172). Conversely, interventions that modify food environments, such as increasing the availability and quality of plant-based options or integrating them into routine settings, have been shown to shift both acceptance and consumption over time (173, 174). These findings underscore that taste is not only individually experienced but structurally mediated.

This structural dimension also brings into focus issues of power and responsibility. Policies that emphasize individual consumer choice without addressing systemic drivers risk obscuring the roles of institutions, markets, and governance in shaping food systems (175–181). Practice-based and sociological perspectives therefore challenge behavior-change models that rely primarily on information provision or nudging, arguing instead for interventions that transform the material and social infrastructures of consumption (182–186).

Taken together, these insights reveal both complementarities and tensions across disciplines. Sensory and consumer sciences offer methodological rigor and conceptual differentiation but risk decontextualizing taste. Sociological and practice-based approaches foreground context, embodiment, and structure but may lack the precision needed for measurement and intervention design. The coexistence of multiple definitions, particularly the inconsistent use of constructs such as liking and preference, further complicates the synthesis of evidence and the development of effective strategies.

These tensions point to a critical gap: the absence of an integrative framework that can reconcile the physiological, experiential, and structural dimensions of taste while maintaining conceptual clarity. Addressing this gap is essential for advancing both research and practice, particularly in the context of sustainable food consumption, where shifting behavior requires aligning sensory appeal with social practices and structural conditions

## 6 Taste in behavior-change models: toward an interdisciplinary framework

Several attempts have been made to integrate disciplinary insights into broader models of behavior and behavior change. However, a consistent limitation across these frameworks is that they tend to operationalize behavior at a relatively abstract level, which can obscure how domain-specific determinants such as taste function in real-world food decisions (see Table 4). As a result, while these models are widely used, their explanatory power varies depending on how well they capture context-dependent and embodied drivers of behavior.

Behavior-change theories (51, 61, 117, 118, 187–190), have been highly influential in explaining individual decision-making processes, particularly through constructs such as attitudes, self-efficacy, and intention formation. However, empirical research shows that these models often struggle to account for the well-documented intention–behavior gap, particularly in food choice contexts where structural constraints, habitual routines, and sensory expectations intervene (191–197). For example, consumers may express positive intentions toward plant-based diets in survey-based Theory of Planned Behavior studies, yet still select meat-based meals in real-world cafeteria or supermarket settings, highlighting a mismatch between stated motivation and situated practice (198, 199).

To address these limitations, broader ecological and multi-level perspectives have been introduced. The Social Ecological Model (SEM) (56, 200) explicitly situates behavior within nested levels of influence, including individual, interpersonal, organizational, community, and policy environments. Empirical applications of SEM in dietary research demonstrate that food choice is shaped not only by individual preferences but also by school food environments, workplace catering systems, retail availability, and national policy frameworks (201–208). However, while SEM improves structural sensitivity, it remains relatively underspecified regarding how sensory and affective determinants, such as taste perception and pleasure, operate across levels.

A similar multi-level integration is offered by the COM-B model (21), which conceptualizes behavior (B) as the interaction between capability (C), opportunity (O), and motivation (M). COM-B has been widely applied in dietary interventions, including school-based nutrition programs, food labeling strategies, and workplace behavior-change initiatives, where it is often used to diagnose barriers and design targeted interventions (e.g., distinguishing lack of access from lack of knowledge or low motivation) (201, 208). Despite this operational usefulness, COM-B typically treats taste implicitly, most often as a component of “motivation” or “preference,” rather than as a multidimensional construct shaped by sensory physiology, learning, and cultural meaning.

In practice, this leads to a conceptual compression: taste becomes reduced to hedonic preference or attitude, despite evidence that it operates across sensory exposure, emotional learning, and social normalization. This limitation is particularly evident in intervention research where “taste” is inferred rather than measured directly, for example through willingness-to-try scales, hedonic liking ratings, or self-reported preference measures in discrete choice experiments. Such operationalizations capture evaluative outcomes but often fail to distinguish between sensory perception (e.g., bitterness detection), learned liking (e.g., repeated exposure effects), and social desirability effects in reporting).

Similar simplifications appear across other dominant frameworks, including the Theory of Planned Behavior, the Health Belief Model, Social Cognitive Theory, Practice Theory, and Nudge/Choice Architecture approaches. Across these models, taste is frequently operationalized as an attitude, a barrier, or an outcome of reinforcement rather than as a structured, multidimensional phenomenon. For instance, in nudge-based cafeteria studies, taste is often indirectly manipulated through labeling or presentation without theorizing how sensory expectation, cultural familiarity, and emotional association jointly shape acceptance (209, 210).

This reduction contrasts with empirical intervention research showing that explicitly targeting taste can significantly alter food choice behavior. For example, Turnwald et al. (211) demonstrated in large-scale dining experiments that indulgent, taste-focused menu descriptions (e.g., *“twisted citrus-glazed carrots”*) increased vegetable selection compared to health-framed labels. Similar findings from supermarkets and digital food environments show that sensory-rich language (e.g., *“rich,”* “*juicy”* or *“creamy”* attributes*)* increases both attention and purchase likelihood for plant-based alternatives. These interventions operationalize taste through controlled framing manipulations, typically measured via sales data, selection rates, or click-through behavior (212–214).

However, while effective, such approaches often treat taste as an externally adjustable lever rather than a socially and biologically embedded process. Sensory and expectation studies further demonstrate that labeling, reformulation, and contextual cues can shift perceived flavor and hedonic evaluation (35), often measured through blinded taste tests, hedonic rating scales, and controlled sensory panels. Yet even these experimental designs tend to isolate sensory response from its broader social and cultural context, limiting ecological validity.

From a sustainability perspective, dominant food system frameworks primarily emphasize health and environmental outcomes, focusing on nutritional adequacy, emissions reduction, and resource efficiency (3, 4, 215). While methodologically robust, these approaches often treat taste as secondary or instrumental, assuming that behavioral change will follow from information, availability, or substitution effects. This creates a gap between sustainability metrics and lived eating experience, where pleasure, familiarity, and cultural meaning remain central drivers of consumption.

A growing body of applied research suggests that more context-sensitive interventions can partially address this gap. Studies on institutional food environments show that repeated exposure in canteens and public kitchens can shift preferences over time by normalizing plant-based or climate-friendly meals (216, 217). Empirical evaluations of such settings indicate that familiarity, routine exposure, and social visibility can increase acceptance even in the absence of explicit motivational interventions (217–223). These findings can be directly compared with labeling studies such as Turnwald et al. (211), suggesting that both informational framing and environmental normalization operate through different but complementary mechanisms: one primarily cognitive and attentional, the other structural and habituate.

These perspectives reveal a persistent analytical gap: although taste is repeatedly identified as central to food choice, it is rarely conceptualized in a way that integrates sensory physiology, emotional learning, and social practice within behavior-change frameworks. Instead, it is fragmented across constructs such as preference, attitude, or hedonic response, depending on disciplinary tradition. This fragmentation limits the ability of existing models to fully explain dietary behavior in real-world settings. This once more highlights the need for a more integrative framework that can connect behavioral, sociocultural, and structural perspectives without reducing taste to a single level of explanation. Such a framework should position taste simultaneously as a sensory phenomenon, an emotional process, and a relational practice embedded in food environments. In doing so, it allows taste to be treated not only as a barrier to sustainable diets but also as a modifiable and productive resource for dietary transition strategies.

## 7 Discussion: toward an interdisciplinary understanding of taste in sustainable food consumption

This discussion integrates the previously presented insights from the conceptual analysis (sections 4–5-6) to address a central question: why does taste remain underused in sustainability interventions despite being a primary driver of food choice? We argue that this gap stems from a misalignment between how taste is conceptualized (fragmented across disciplines) and how it is operationalized (simplified in behavior-change models). Bridging this divide requires reconceptualizing taste as an embedded, multi-level phenomenon and repositioning it as a strategic lever in dietary transitions.

### 7.1 Conceptual divergences and integrative prospects in the study of taste

The review reveals not just differences, but systematic tensions across disciplines that explain why taste is difficult to integrate into sustainability frameworks.

First, there is a level-of-analysis tension. Sociological and anthropological approaches conceptualize taste as socially structured and embedded in practices (Section 4.1), whereas sensory and consumer sciences treat it as an individual, measurable response (Section 4.2). This divide is not merely terminological, it leads to different intervention logics. One prioritizes structural change (e.g., food environments), the other targets individual perception (e.g., reformulation, labeling). As shown in Section 6, existing behavior-change models such as COM-B and SEM attempt to bridge levels, yet fail to integrate taste across them, instead implicitly assigning it to “motivation” or “preference.” This results in what can be interpreted as a conceptual compression of taste, where its sensory, emotional, and social dimensions are collapsed into a single variable.

Second, there is a measurement–meaning tension. Section 5 demonstrates that constructs such as liking, preference, and taste are inconsistently defined, while Section 6 shows that empirical studies often rely on proxy measures (e.g., hedonic ratings, willingness-to-try). While these enable quantification, they capture evaluative outcomes rather than underlying processes, making it difficult to compare findings across disciplines or translate them into real-world interventions.

Third, there is a context gap between experimental and lived experience. As evidenced across Sections 4.2 and 6, laboratory-based sensory studies and controlled behavioral interventions often isolate taste from its social and material context. In contrast, sociological and practice-based research shows that taste evolves through repeated exposure, social interaction, and environmental structuring. The result is a persistent disconnect between what works experimentally (e.g., sensory framing) and what sustains behavior in practice (e.g., normalization in food environments).

Taken together, these tensions explain why taste is simultaneously recognized as central yet under-theorized and underutilized in sustainability research and interventions.

### 7.2 Reconceptualizing taste: the concept of sustainable taste

To address these gaps, we propose the concept of “sustainable taste” as a unifying framework that integrates sensory, psychological, and sociocultural dimensions while remaining applicable to intervention design. We define sustainable taste as:

*A situated, socially and culturally embedded experience of food that combines sensory pleasure, emotional meaning, and relational context, and that motivates, enables, and normalizes sustainable food practices in everyday life.*

This reconceptualization directly responds to the limitations identified across Sections 4–6 in three key ways:

First, it resolves the level-of-analysis tension by positioning taste as inherently multi-level. The three components, Sensation, Emotion, and Relation, map onto physiological processes, affective drivers, and social-structural contexts, respectively. Rather than assigning taste to a single domain (e.g., motivation), this framework conceptualizes it as operating across capability, opportunity, and motivation simultaneously. Second, it addresses the measurement–meaning gap by distinguishing between different dimensions of taste and their corresponding empirical operationalizations. For example: Sensation can be captured through sensory tests and perceptual measures, Emotion through hedonic ratings and affective responses, and Relation through observational, ethnographic, and environmental indicators. This multidimensional structure allows existing methodologies (Section 5 and 6) to be reinterpreted as partial measures of a broader construct, rather than isolated proxies. Third, it bridges the gap between experimental and real-world contexts by embedding taste within practices and environments. Evidence from institutional food settings (Section 6) demonstrates that repeated exposure, social visibility, and routine participation can reshape preferences over time. Within the sustainable taste framework, such findings are not peripheral but central: they illustrate how taste is formed through interaction between individuals and environments, rather than being a fixed attribute.

Importantly, this reconceptualization shifts the role of taste in sustainability transitions. Rather than treating taste as a barrier to overcome, it becomes a modifiable and productive resource that can be actively shaped through: sensory design (e.g., flavor optimization, framing), emotional engagement (e.g., nostalgia, pleasure), and relational interventions (e.g., communal eating, food environments). This integrative perspective not only synthesizes disciplinary insights but also provides a conceptual foundation for operational frameworks, such as the SER extension of COM-B developed in the following section.

This framework also has direct implications for behavior-change practice. By structuring taste into sensory, emotional, and relational components, the SER framework enables researchers and practitioners to more precisely diagnose which dimensions of taste act as barriers or facilitators in specific contexts. For example, low acceptance of plant-based foods may stem from sensory unfamiliarity (Sensation), negative expectations or lack of pleasure (Emotion), or limited exposure within social and institutional settings (Relation). This differentiation allows interventions to be more effectively targeted: sensory education and repeated exposure can build familiarity, taste-focused communication can enhance hedonic appeal, and modifications to food environments (e.g., canteens, retail settings) can normalize sustainable options. In this way, sustainable taste moves from a descriptive concept to a practical tool for designing, tailoring, and evaluating behavior-change interventions.

### 7.3 Integrating sustainable taste into a behavior-change framework: COM-B + SER

**Figure 1:**
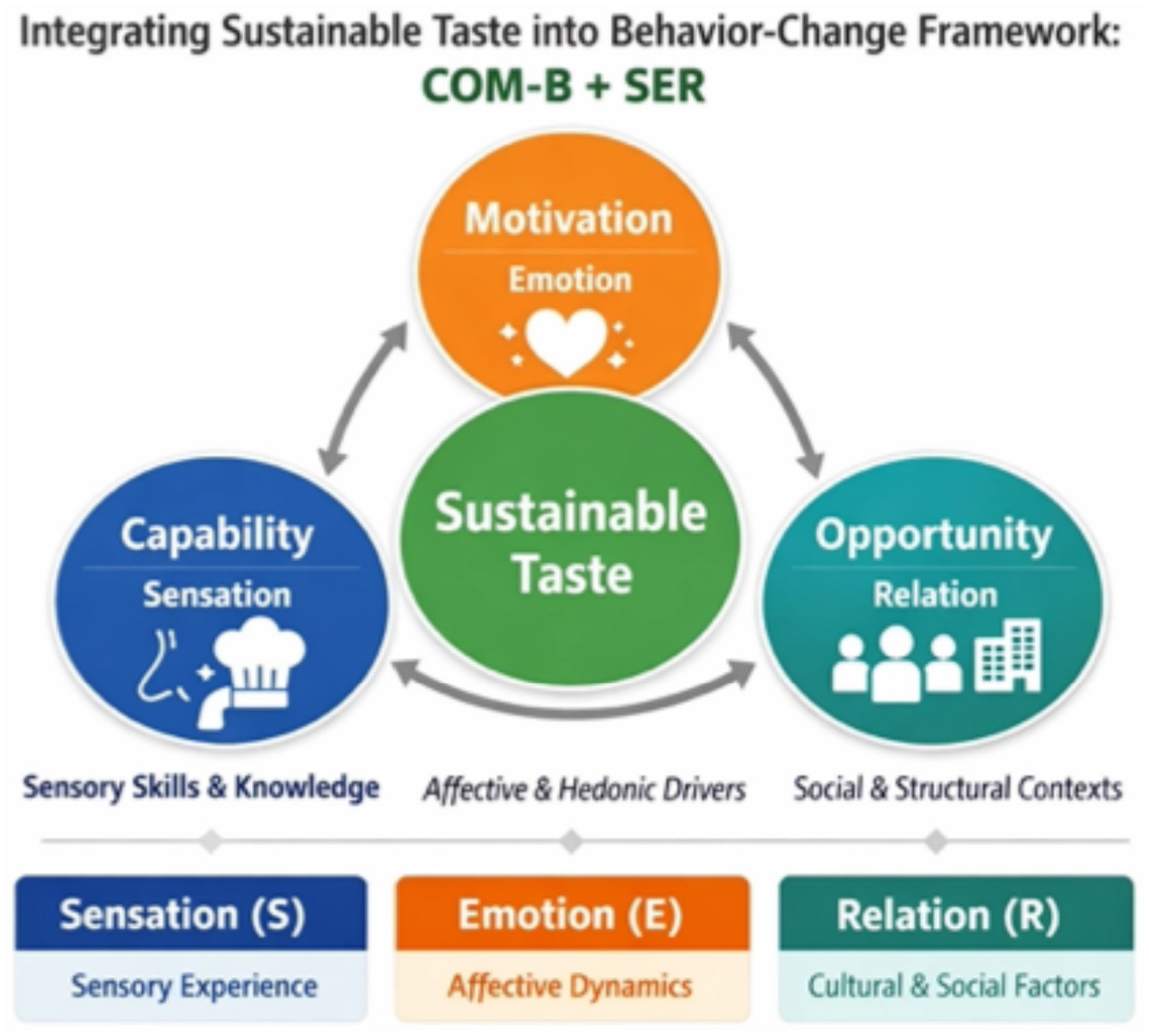
Integration of sustainable taste into the COM-B model using the SER (Sensation-Emotion-Relation) framework.

To connect the concept of sustainable taste to established behavior-change theory, we draw on the COM-B model (21) as an organizing framework. COM-B conceptualizes behavior as a function of capability, opportunity, and motivation, and is widely used in dietary behavior research. While COM-B offers a flexible umbrella model, it rarely captures the sensory, emotional, and relational dimensions of taste explicitly. To address this limitation, we extend COM-B through the SER framework (Sensation–Emotion–Relation), which operationalizes sustainable taste across research, policy, and intervention contexts (See Figure 1).

Importantly, the SER framework can also be operationalized as a diagnostic and design tool. In practice, it enables researchers and policymakers to identify where barriers to sustainable eating are primarily located: whether in sensory capability (S), emotional-motivational dynamics (E), or relational-structural conditions (R). This mapping allows interventions to be more precisely targeted rather than treating “taste preference” as a single, undifferentiated barrier.

#### 7.3.1 Capability and sensation

Capability refers to individuals’ physical and psychological capacity to engage in a behavior. Taste intersects with capability insofar as taste capacities are shaped by sensory acuity, familiarity with flavors, culinary skills, and food-related knowledge. The sensation component of SER highlights how flavor literacy, hedonic sensitivity, and sensory learning influence individuals’ ability to prepare, appreciate, and enjoy sustainable foods. Interventions that enhance sensory capability, such as sensory education programs, tastings, and cooking workshops, can therefore be used as practical entry points for intervention design, particularly for increasing acceptance of novel sustainable foods (e.g., plant-based proteins, seasonal vegetables). Socio-economic capabilities, including income, education, and access to infrastructure, further shape feasibility. Thus, capability is not only individual but also socially and structurally conditioned.

#### 7.3.2 Motivation and emotion

Motivation encompasses the psychological processes that energize and direct behavior. Taste is deeply intertwined with motivation, as food choices are strongly driven by pleasure, comfort, familiarity, and affective meaning. The emotion component of SER emphasizes how affective responses, such as pleasure, nostalgia, guilt, or comfort, interact with identity, values, and cultural repertoires to shape food motivations. From an intervention perspective, SER suggests that strategies focusing only on informational or normative appeals may be insufficient; instead, leveraging positive affect and hedonic expectation can enhance uptake of sustainable diets. Emotional mapping of food practices can thus support the design of culturally resonant interventions that align sustainability with pleasure and identity.

#### 7.3.3 Opportunity and relation

Opportunity refers to the external conditions that enable or constrain behavior. Taste intersects with opportunity through social, cultural, structural, and material environments that shape exposure to foods and normalize certain practices. The relational component of SER highlights how cultural norms, networks, infrastructures, and policy environments structure opportunities for sustainable eating. This also provides a policy-relevant diagnostic lens, allowing decision-makers to distinguish between individual-level preference barriers and structural constraints such as availability, affordability, or institutional food provision. Accordingly, interventions may range from modifying food environments (e.g., cafeteria redesign, procurement standards) to strengthening social exposure through communal eating initiatives and public tastings.

### 7.4 Implications for research, policy, and interventions

Integrating taste into each element of the COM-B model ensures that interventions are grounded in real-world eating practices rather than assuming that sustainable foods are inherently unappealing. This perspective also informs practice theory, social marketing, and food policy by positioning taste as central to everyday food practices. By operationalizing sustainable taste through the SER framework, taste shifts from being treated as a residual factor or barrier to becoming a strategic lever for dietary transitions.

National campaigns can combine taste-focused messaging with communal tasting experiences; institutional catering standards can prioritize flavor alongside nutrition and sustainability; and co-creation with communities can align interventions with local tastes and cultural norms. Overall, this integrative discussion underscores that sustainable eating is not only a rational or ethical choice but also a sensory, emotional, and relational experience.

Future research should empirically test the SER framework as a diagnostic tool, examining how effectively it identifies dominant barriers across different populations and contexts. In particular, comparative intervention studies are needed to assess whether SER-informed interventions lead to improved dietary outcomes compared to standard COM-B-based approaches. Further work should also explore how sensory, emotional, and relational dimensions interact dynamically over time, and how these interactions differ across cultural and socio-economic settings. Finally, methodological development is needed to translate SER categories into standardized measurement instruments suitable for both qualitative and quantitative research designs.

Foregrounding taste as a multidimensional driver of behavior enhances the appeal, cultural resonance, and long-term effectiveness of interventions aimed at transforming food systems toward sustainability.
